# Enhancing the Formation and Stability of Oil-In-Water Emulsions Prepared by Microchannels Using Mixed Protein Emulsifiers

**DOI:** 10.3389/fnut.2022.822053

**Published:** 2022-05-27

**Authors:** Yan Jiao, Yuntai Zhao, Ying Chang, Zhaoxiang Ma, Isao Kobayashi, Mitsutoshi Nakajima, Marcos A. Neves

**Affiliations:** ^1^Graduate School of Life and Environmental Sciences, University of Tsukuba, Tsukuba, Japan; ^2^College of Food and Biological Engineering, Qiqihar University, Qiqihar, China; ^3^Biobased Chemistry and Technology, Wageningen University and Research, Wageningen, Netherlands; ^4^Alliance for Research on Mediterranean and North Africa (ARENA), University of Tsukuba, Tsukuba, Japan

**Keywords:** formation, stability, microchannel, monodisperse emulsion, mixed protein

## Abstract

Although natural emulsifiers often have many drawbacks when used alone, their emulsifying ability and stability can usually be improved unexpectedly when used in combination. In this study, monodisperse emulsions stabilized by combining two natural protein emulsifiers, i.e., whey protein isolate (WPI) and sodium caseinate (SC), in different proportions were prepared using microchannel (MC) emulsification. The influences of temperature, pH, ionic strength, and storage time on the microstructure and stability of the emulsions were examined. Analysis of the microstructure and droplet size distribution revealed that the WPI-, SC-, and mixed protein-stabilized emulsions exhibited uniform droplet distribution. The droplet size and ξ-potential of the MC emulsions stabilized by mixed protein emulsifiers were higher than those of the emulsions stabilized by WPI or SC separately. The emulsions stabilized by the two types of proteins and mixed emulsifiers had better stability under high salt concentrations than the synthetic emulsifier Tween 20. WPI-SC-stabilized emulsions were more resistant to high temperatures (70–90°C) and exhibited excellent stabilization than those stabilized by WPI and SC, which was attributed to the more sufficient coverage provided by the two types of protein emulsifier layers and better protein adsorption at the oil-water interface. These results indicate that WPI-SC is a potential stabilizer for MC emulsion requirements. This study provides a basis for the formulation of monodisperse and stable natural emulsion systems.

## Graphical Abstract

Formulation of monodisperse emulsions stabilized by mixed protein emulsifiers using microchannel emulsification. 
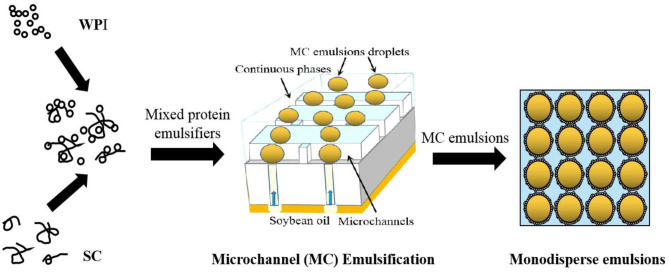

## Introduction

Currently, food emulsions are mainly prepared using high-pressure homogenizers, microfluidizers, colloid mills, various rotor-stator homogenizers, and ultrasonic techniques. These techniques have disadvantages, such as heat generation due to shear forces, the production of emulsion droplets with polydispersity, lower emulsion stability, and lower encapsulation efficiencies (1–3). Compared with conventional emulsification, microfluidic techniques, such as membrane emulsification and microchannel (MC) emulsification, have been developed that can produce monodisperse emulsions, which can control the properties of emulsions, especially their stability (4, 5).

The MC emulsification technique is more effective than the use of conventional devices. MC emulsification can prepare monodispersed emulsions with better stability, a narrower droplet size distribution, and lower energy input. MC emulsification has been also successfully applied for preparing simple and multiple emulsions, microspheres, and microcapsules to encapsulate numerous functional compounds (6, 7).

Generally, food emulsions are unstable systems that can be broken down under different conditions, such as high temperatures, acidic and alkaline environments, and high ionic strength, among others. Although emulsions prepared by MC emulsification have a monodisperse droplet size distribution, they also break down easily under various food processing and storage conditions (8, 9). Therefore, the appropriate emulsifier is very important for the stability of the emulsion.

Many natural emulsifiers have recently been used to prepare stable MC emulsions, and promising results have been obtained; for example, proteins, polysaccharides, phospholipids, and some plant extracts have been used to prepare MC emulsions (10–12). Food proteins are used as emulsifiers in a variety of industries because of their ability to facilitate emulsion formation and improve emulsion stability. Proteins separated from bovine milk are the major proteins that have been used to successfully prepare MC emulsions, especially, whey protein isolates (WPIs), β-lactoglobulin, sodium caseinate (SC), and bovine serum albumin (BSA). For example, Khalid et al. (13) studied the formulation and characterization of monodisperse oil-in-water (O/W) emulsions prepared by synthetic and natural emulsifiers using straight-through MC emulsification (13). O/W emulsions encapsulating astaxanthin extracts (AXT) were successfully formulated by polyglycerol fatty acid ester emulsifiers (MO-7S and ML-750) and SC. The monodispersity of AXT-loaded oil droplets was well maintained during MC emulsification, the synthetic emulsifier achieved better emulsification, and the natural SC caused the wetting of the MCs and exhibited a high polydispersity index (PDI). Charcosset et al. (2) investigated the formulation and stability characteristics of monodisperse O/W emulsions encapsulating fucoxanthin using straight-through MC emulsification stabilized with modified lecithin (ML), WPI, and Tween20 (Tw20). The results showed that emulsions prepared by MC emulsification exhibited good physical and chemical stability during the long-term storage experiments at low temperatures (14). Alliod et al. (3) prepared O/W emulsions stabilized by BSA by straight-through MC emulsification or homogenization and investigated how the stability of the prepared emulsions was affected by thermal processing, freeze-thaw treatment, pH adjustment, and NaCl addition. Their results showed that the emulsions prepared by MC emulsification had higher stability under thermal processing than the emulsions prepared by homogenization (15).

Food-grade emulsions prepared with single natural emulsifiers are less stable than those prepared with synthetic emulsifiers; therefore, natural emulsifiers must be optimized to stabilize the emulsion more effectively according to different characteristics (16, 17). Many studies have demonstrated the defects of natural emulsifiers that include their lack of heat resistance, tendency to denature at high salt concentrations, and short storage time. Therefore, natural emulsifiers have been used in combination to improve their functional performance. Some researchers have reported that the formation, stability, and functional performance of emulsions could be enhanced using appropriately mixed emulsifiers. For example, Xu et al. examined the potential of combining two natural emulsifiers, hydrolyzed rice glutelin and quillaja saponin, in the formation and stability of O/W emulsions. Their results showed that the mixed emulsions had better stability under high salt levels and temperatures at pH 7, which was attributed to the thicker interfacial layer causing stronger steric repulsion (18). Xue and Zhong studied the formation of thymol nanodispersions by blending lecithin and gelatin, which formed a synergistic surface to prevent particle size changes due to coalescence and Ostwald ripening, maintained the particle dimensions during storage at a neutral pH, and improved the storage stability of the dispersion (19). Boonlao et al. evaluated the stability of an emulsion system stabilized by WPI (2–5 wt%) and xanthan gum (XG) (0.25 and 0.5 wt%) and found that the WPI-XG-stabilized emulsion exhibited higher astaxanthin stability at lower storage temperatures (5, 25, and 37°C), with 10–12% astaxanthin loss over 15 d of storage (20). However, few studies have been conducted on emulsions prepared by MC emulsification with mixed protein emulsifiers.

In this study, we aimed to prepare monodisperse emulsions by MC emulsification stabilized by two different types of protein emulsifiers separately or in combination. We also characterized MC emulsions stabilized by different emulsifiers and analyzed and compared their physical stability.

## Materials and Methods

### Materials

Whey protein isolate was procured from NICHIGA, Japan Garlic Co., Ltd. (Takasaki, Japan). SC, polyoxyethylene (20), sorbitan monolaurate or Tw20, soybean oil, and sodium azide were purchased from Wako Pure Chemical Industries Company (Osaka, Japan). All other chemicals used in this study were of analytical grade. Ultrapure water produced using an Arium^®^ Pro System (Sartorius, Goettingen, Germany) was used to prepare all solutions and emulsions.

### Formulation of Continuous and Dispersed Phases

Continuous phases were prepared by dissolving emulsifiers at different aqueous conditions. Briefly, 1% (w/w) Tw20, WPI, SC, and mixed proteins (0.75% WPI + 0.25% SC, 0.5% WPI + 0.5% SC, and 0.25% WPI + 0.75% SC) were dissolved in Ultrapure water containing 0.02 wt% sodium azide as a preservative with constant stirring for almost 12 h at ambient temperature to ensure complete mixing before using as the continuous phase. Soybean oil was used as the dispersed phase.

### Preparation of MC O/W Emulsions

**Figure 1:**
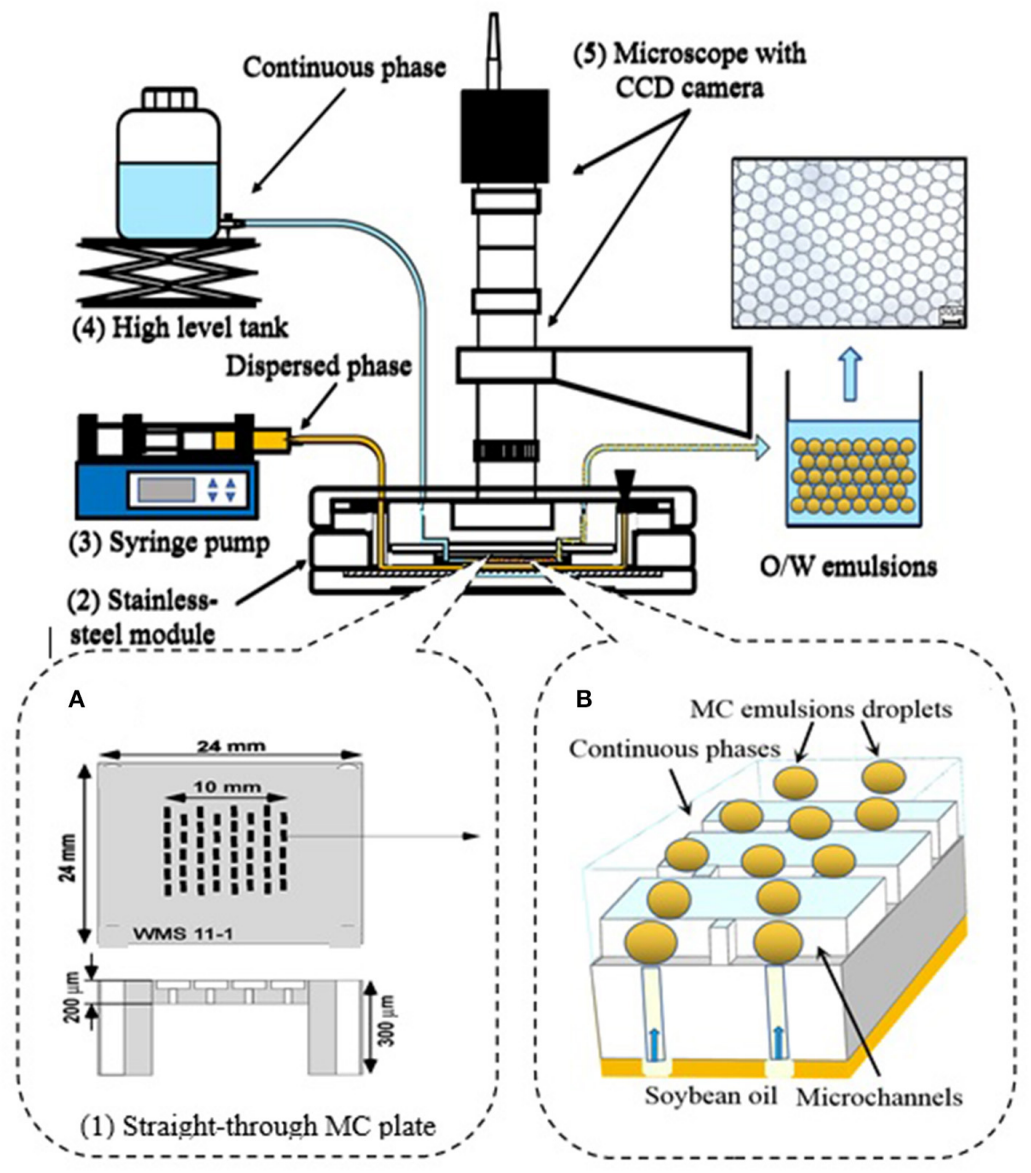
Setup of the microchannel (MC) emulsification instruments. **(A)** Asymmetric straight-through MC array plate. **(B)** MC emulsion formation process in the MC array plate.

Emulsions were prepared by MC emulsification following our previous method, with some modifications (21). Tw20, WPI, SC, and mixed protein emulsions were prepared using MC emulsification experiment instruments with an asymmetric straight-through MC array (Figure 1). The MC emulsification experiment instruments consisted of the following elements: (1) a silicon asymmetric straight-through 24 × 24-mm MC array plate containing 13,752 MCs on an active area of 1 cm^2^ (WMS11-1, EP Tech Co., Ltd., Hitachi, Japan), these MCs were arranged on an active area of 10 mm^2^. The MC array plate was 300-μm thick, but the active area containing MCs was etched to 200 μm (Figure 1A). (2) A stainless-steel module with a liquid chamber to feed the dispersed phase (O/W emulsion). (3) A syringe pump (Model 11; Harvard Apparatus. Inc., Holliston, USA) to feed the continuous phase (outer aqueous phase). (4) A high-level tank for providing the continuous phase. (5) An optical microscope connected to a CCD camera and video system for monitoring droplet generation.

The emulsion preparation process was as follows: the dispersed phase of soybean oil was supplied at a flow rate ranging from 0.8 to 1.0 ml/h. The continuous phase was supplied from an elevated reservoir and flowed through the gap between the MC plate and coverslip. The flow rate of the continuous phase was controlled from 150 to 200 ml/h during droplet generation. The MC emulsification was monitored using a microscope and an image was captured by the CCD camera.

### Droplet Size Measurement of MC Emulsions

The droplet size and size distribution of the O/W emulsions were analyzed using a laser diffraction particle size analyzer (LSI3 320, Beckman Coulter, Brea, CA, USA). The average diameter (d_4,3_) represents the droplet size (22).

### ξ-Potential and Z-Average Diameter (dz) Analysis

The ξ-potential of each sample was determined using a Nano-Zetasizer (ZS) instrument (Malvern Instrument Ltd., UK) via the laser Doppler velocimetry technique. A portion of the emulsion was diluted (1:50) using ultrapure water and mixed thoroughly. The diluted sample was then injected into the measurement chamber of the instrument, and a voltage was applied according to the determination procedure. The measurements were conducted at room temperature. The particle size and PDI of the emulsifiers were also determined using the Nano-ZS instrument (23).

### Stability of the Emulsions Prepared by MC

#### Thermal Stability of the MC Emulsions

Six types of diluted MC emulsion samples (5 ml) were transferred into glass test tubes and placed in a water bath for 120 min at a fixed temperature ranging from 30 to 90°C. Next, the emulsion samples were cooled down to room temperature and their average droplet diameter (*d*_4,3_) and coefficient of variation (CV) were determined using a laser diffraction particle size analyzer (24).

#### pH Stability of the MC Emulsions

Five milliliters of diluted emulsions were prepared at different pH levels (3–9) by adjusting the samples with 0.1 M HCl or NaOH. The samples were stored at 25°C for 24 h before analysis. The *d*_4,3_ and CV of the emulsions at different pH levels were then analyzed (25, 26).

#### Ionic Strength Stability of the MC Emulsions

One milliliter of emulsion samples was added to the 5-ml NaCl solutions to prepare samples with the desired final salt concentrations (0, 50, 100, 150, and 200 mM). The samples were then stored at 25°C for 24 h prior to analysis. The *d*_4,3_ and CV of the emulsions under different salt concentrations were then analyzed (27).

#### Storage Stability of the MC Emulsions

The emulsions (neutral pH, no salt added) were stored under darkness at different temperatures (25 or 50°C) for up to 15 d. The *d*_4,3_ of the emulsions was analyzed throughout the storage period to monitor their physical stability (28).

## Results and Discussion

### Preparation of MC Protein Emulsions by Different Emulsifiers

**Figure 2:**
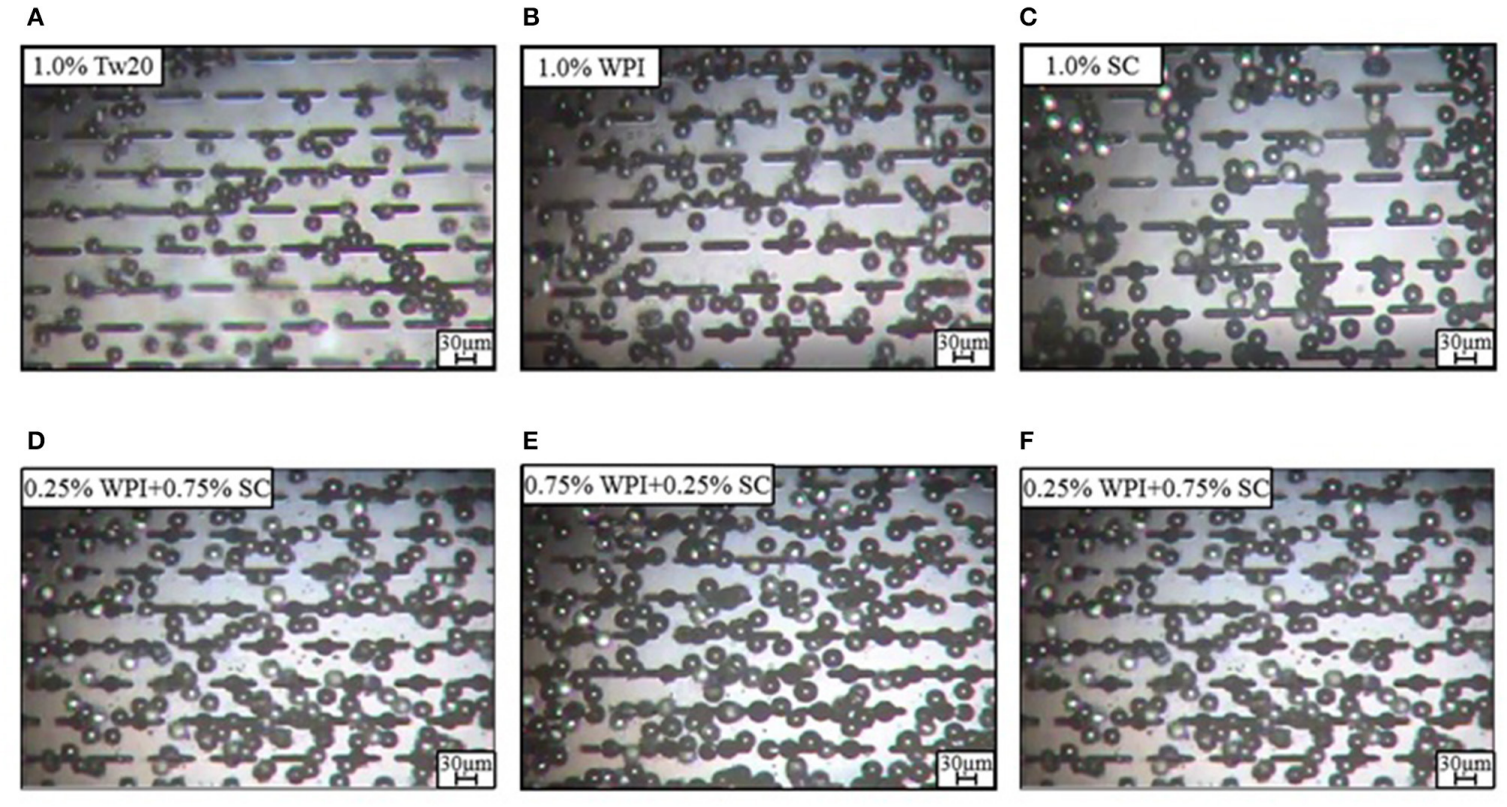
Droplet formulation of MC emulsions with the different types of emulsifiers.

We successfully prepared six types of MC emulsions using WPI, SC, and mixed protein emulsifiers with three different concentrations and compared them to Tw20. Based on the micrographs of the emulsions prepared by MC emulsification (Figures 2A–F), it was found that all emulsifiers could form soybean oil-based O/W emulsions from the MC tip. Stable emulsion droplet generation was observed in all working MCs, and all of the emulsifiers generated emulsions with a regular size and uniform distribution, indicating that WPI, SC, and mixed protein emulsifiers were suitable for preparing MC emulsions.

### Droplet Size Analysis of the Emulsions

**Figure 3:**
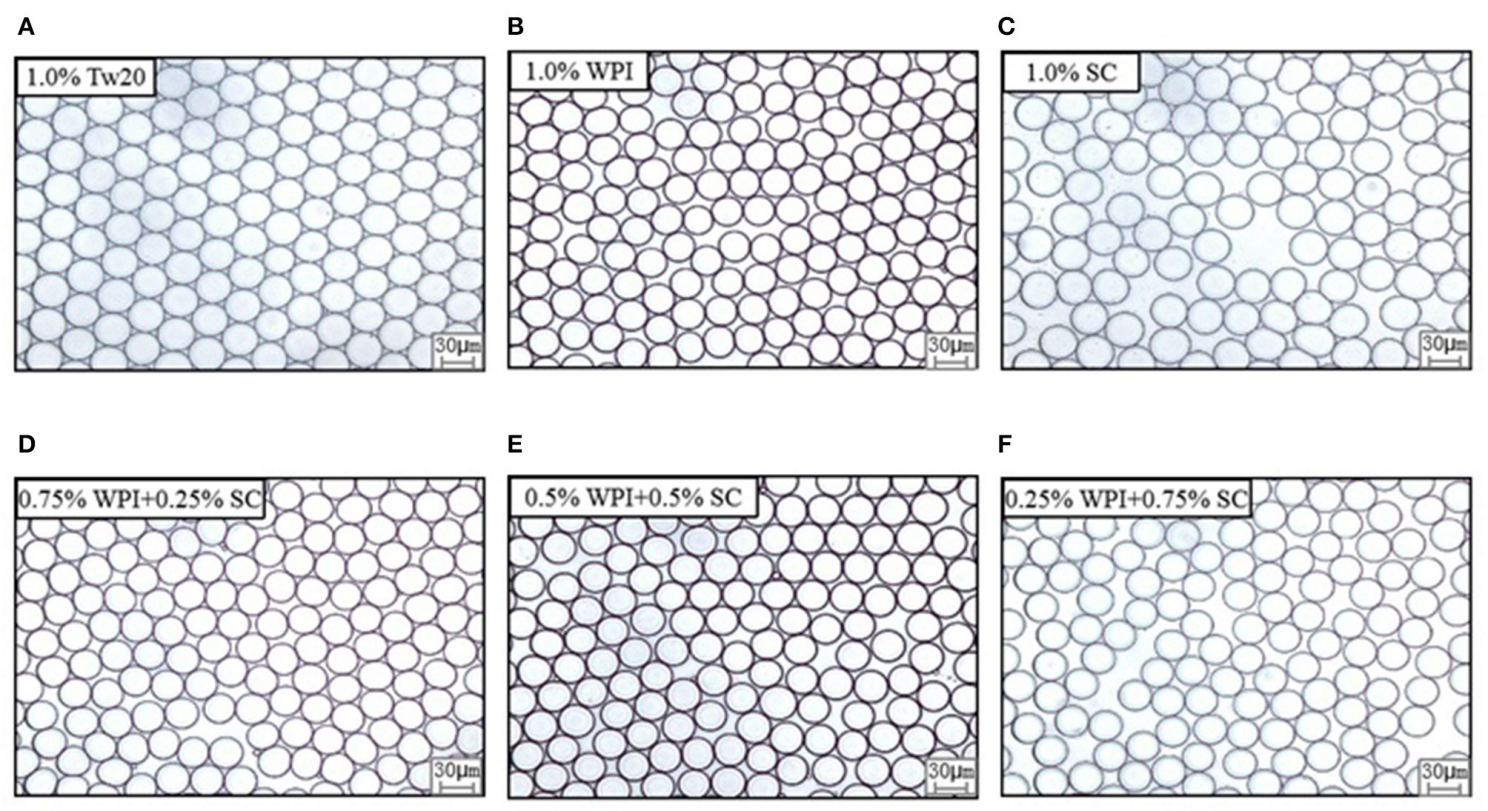
Effects of emulsifier type on the droplet's morphology and distribution of MC emulsions under the microscope.

**Figure 4:**
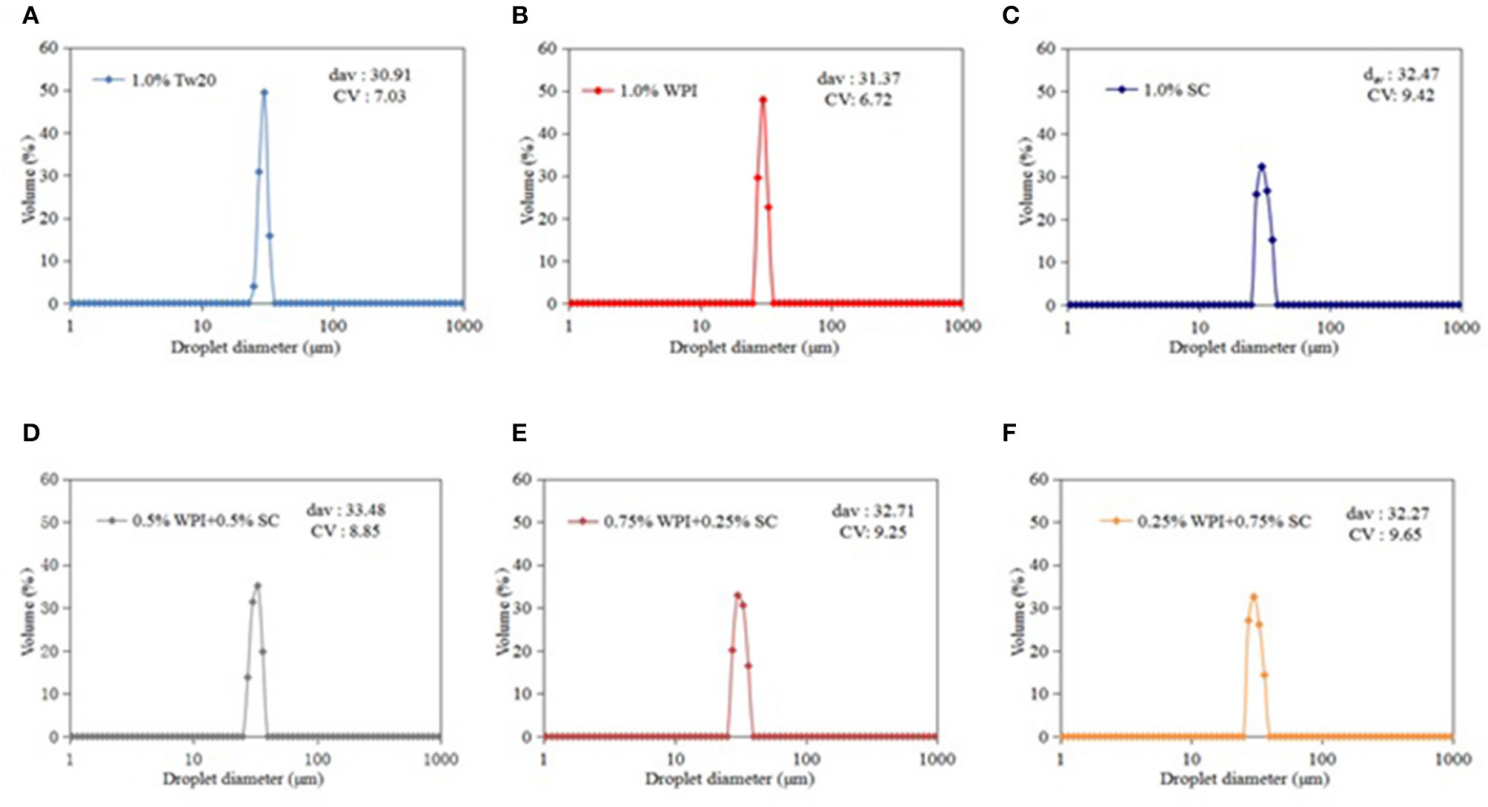
Droplet size distribution of the microchannel (MC) emulsions stabilized by different emulsifiers.

The MC emulsions were observed using an optical microscope (Leica DFC7000T, Heidelberg, Germany) (Figures 3A–F). The emulsion droplets prepared by MC with different types of emulsifiers were uniform and had a diameter of ~30 μm. The smaller droplet sizes and narrower droplet distribution are presented in Figures 4A–F. All emulsions had narrow droplet size distributions with CVs smaller than 10%, indicating that monodisperse droplets were prepared by MC, and the Tw20 and WPI-based emulsions had a narrower droplet size distribution than the SC-based emulsions.

Figure 4 shows that the MC emulsions stabilized by natural protein emulsifiers have larger average droplet diameters than the Tw20-stabilized emulsion because synthetic emulsifiers have an excellent emulsification ability that allows them to form a thinner emulsion layer on the surface of oil droplets, resulting in the generation of smaller emulsion droplets. However, the droplets of the emulsion produced with mixed proteins were larger than those produced with SC and WPI, which may be because both proteins can be combined with soybean oil when the disordered SC molecules are incorporated with globular WPI; they were both adsorbed at the oil/water interface to form three-phase emulsions. Therefore, the combined effect of mixed emulsifiers and oil droplets may have led to a change in droplet size. The emulsion droplets were more monodisperse (29). Moreover, when the proportion of SC was excessively high, redundant SC cannot effectively bind to the oil surface, resulting in a decrease in the emulsification efficiency of the mixed protein emulsifier. Therefore, the mixed proteins (0.5% WPI + 0.5% SC) achieved a better emulsification ability.

### Z-Average Diameter Analysis of the Emulsifiers

To verify the different emulsifying abilities of the mixed emulsifier, we investigated the properties of the single and mixed emulsifiers, particularly, particle distribution, aggregation, and charge of the mixed protein emulsifier in an aqueous solution, to explore the effect of the mixed emulsifier on the emulsion properties (30).

**Figure 5:**
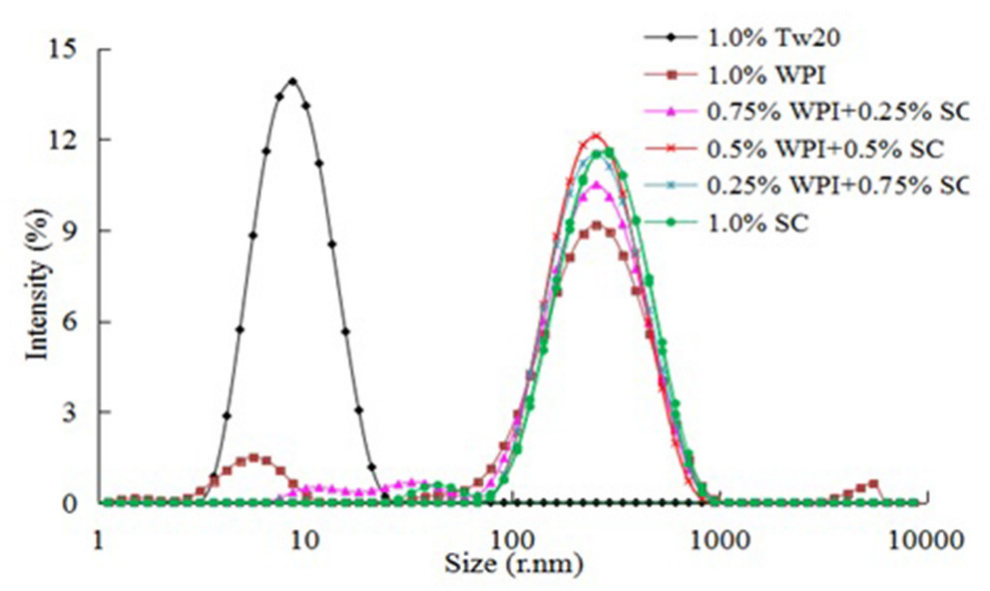
Particle size distribution of the microchannel (MC) emulsions stabilized by different emulsifiers.

As shown in Figure 5, the Tw20-based emulsifier achieved good dispersion (PDI = 0.15 ± 0.018) and a narrow distribution range in water and could easily combine with oil to form an emulsion. The PDI of the 0.5% WPI and 0.5% SC were larger (0.49 ± 0.005 and 0.27 ± 0.007, respectively) as they are both natural milk proteins. Therefore, they had a slightly larger particle size and poorer performance in the water system than Tw20 and wider size distribution. However, when 0.5% WPI was combined with SC, they exhibited uniform distribution in water, a narrower size distribution, and a smaller PDI value (PDI 0.32 ± 0.002). Therefore, the mixed emulsifier could combine well with a large amount of oil to form a relatively stable MC emulsion.

### ξ-Potential Analysis of the Emulsifiers and MC Emulsions

**Figure 6:**
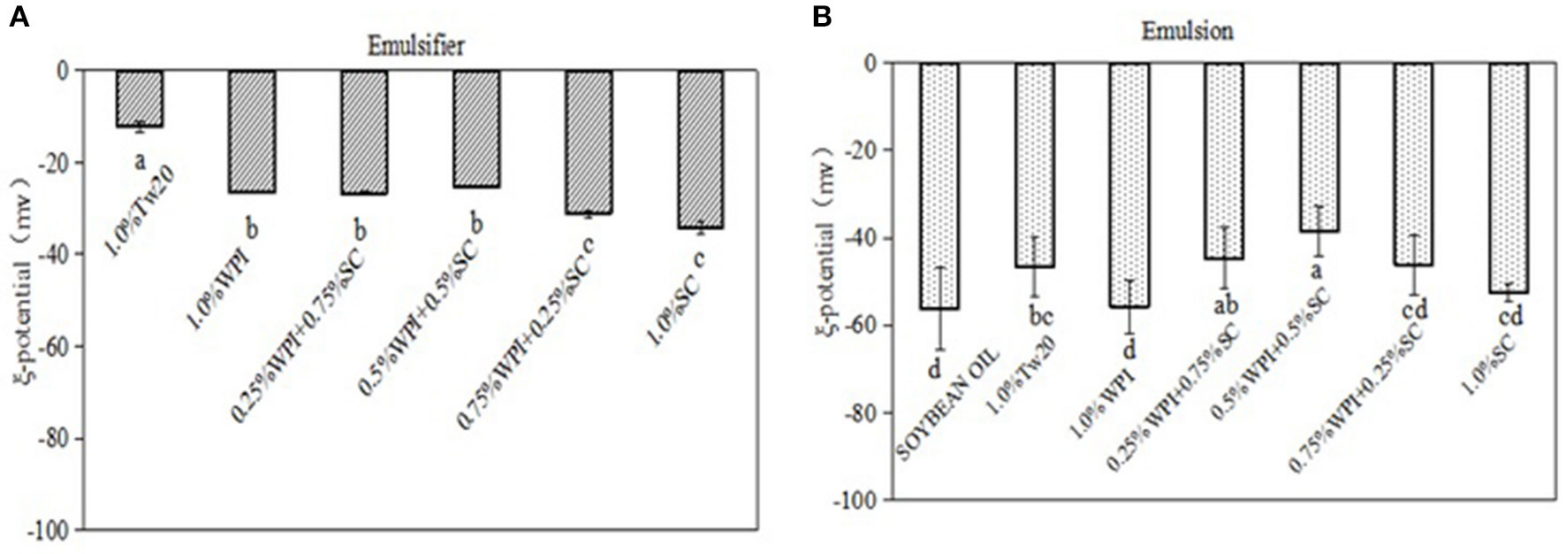
Comparison of the ξ-potentials for different emulsifier solutions and emulsions. Different superscript letters in the columns indicate significant differences (*p* < 0.05).

To study the role of the electrostatic interactions of emulsifiers on the formation and stability of emulsions, the ξ-potentials of different emulsifiers and their emulsions were measured (Figure 6). The results showed that the combination of the two proteins formed mixed emulsifiers and led to a change in the ζ-potential, indicating that the charges of the WPI and SC decreased when they were mixed thoroughly, and the ζ-potential of the mixed protein was slightly increased. After combining with the soybean oil, the ζ-potential of the emulsions was decreased due to the negative potential of the soybean oil. The emulsions stabilized by WPI and SC had lower ζ-potential than those stabilized with Tw20; however, the ζ-potentials of the emulsions stabilized by the mixed emulsifiers were higher, especially the emulsions stabilized by 0.5% WPI and 0.5% SC. The decrease in the ζ-potential may be due to the decrease in the electrical charge of the mixed proteins and the adsorption of mixed proteins molecules that shifted the shear plane away from the surface of the droplets. Therefore, the mixed protein emulsifier could form more stable emulsions (18).

### Stability of the MC Emulsions

#### Effect of Temperature on the Stability of the MC Emulsions

**Figure 7:**
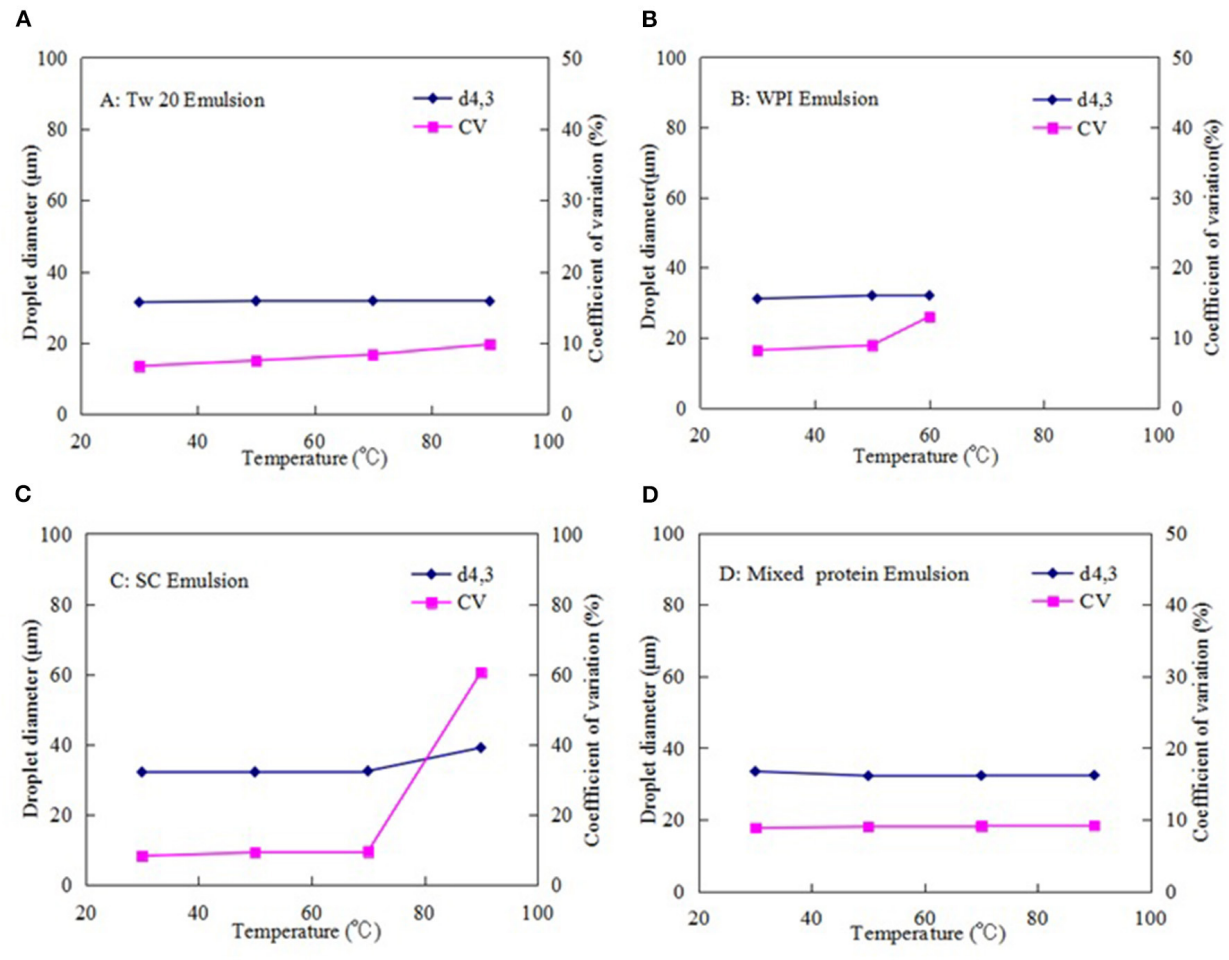
Effect of temperature on the stability of the microchannel (MC) emulsions stabilized by: **(A)** Tween20 (Tw20), **(B)** whey protein isolate (WPI), **(C)** sodium caseinate (SC), and **(D)** mixed proteins.

Figures 7A–D presents the thermal stability of each emulsion prepared with Tw20, WPI, SC, and mixed proteins (0.5% WPI + 0.5% SC) by MC in the temperature range of 30–90°C. The droplets of all MC emulsions were spherical at 30°C, and the droplet diameter was maintained at ~30 μm. As the temperature increased, there was no significant change in the Tw20-stabilized MC emulsion below 90°C, suggesting that the Tw20-stabilized MC emulsions had good thermal stability under short heating conditions. However, when WPI was used as an emulsifier, the shape and diameter of the droplets in the emulsion were changed easily in the temperature range of 50–60°C; when the temperature reached 70°C, the droplets coalesced, and the emulsion aggregated into a gel at 90°C. These results showed that the thermal denaturation of adsorbed globular proteins at high temperatures led to the exposure of amino acid residues originally located within their hydrophobic interiors (31).

Similarly, the shape and diameter of the SC emulsion droplets were changed greatly above 70°C. Therefore, the MC emulsions stabilized by casein exhibited poor thermal stability and were unstable above 70°C for the protein denaturation of WPI. When the mixture of SC and WPI (0.5% SC + 0.5% WPI) was used as an emulsifier, the stability of the emulsion was improved; i.e., there was little change in droplet shape and size below 70°C and even at 90°C, a small amount of coalescence was occurred, even at 90°C, and there was no notable increase in the droplet size and CV. This may be because the mixed protein emulsifier could reduce the interfacial tension and form a layer at the interface of soybean oil and aqueous phase with sufficient coverage, preventing droplets from coalescing or flocculating via steric and electrostatic stabilization mechanisms and the proteins crosslinking on the droplet at high temperature. This can inhibit the susceptibility of the emulsion to heat-induced destabilization, reducing the damage to the emulsion droplets caused by the external temperature (29). These results are consistent with those in previous literature, which reported that a mixed casein and WPI emulsifier can better control the heat-induced aggregation of WPI and improve the stability of the emulsion at high temperatures than using WPI or casein separately (32).

#### Influence of pH on Emulsion Stability

**Figure 8:**
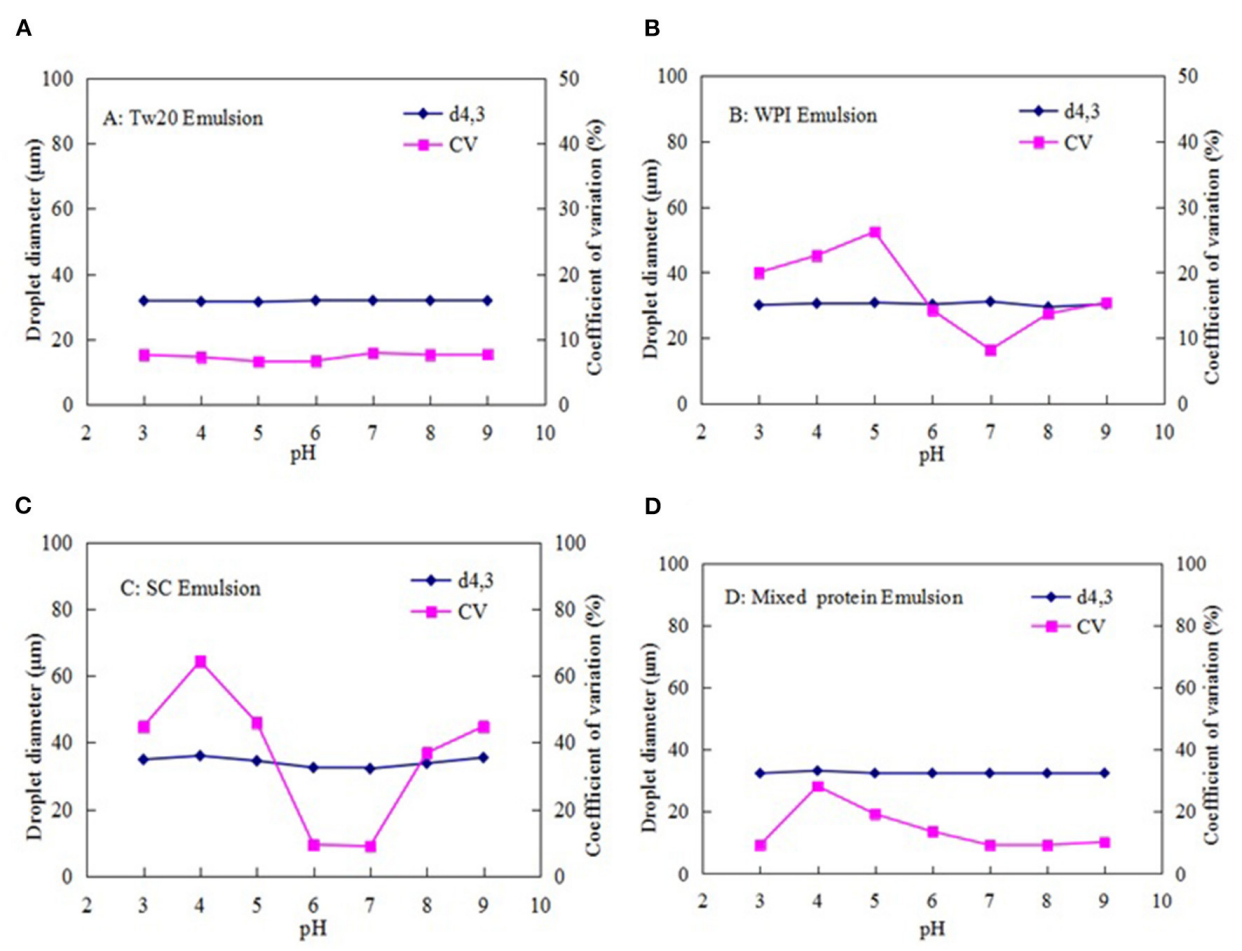
Effect of pH on the stability of the MC emulsions stabilized by **(A)** Tw20, **(B)** WPI, **(C)** SC, and **(D)** mixed protein.

The stability of the MC emulsions prepared with Tw20, WPI, SC, and the mixed proteins was investigated at pH 3.0–9.0 (Figure 8). Both low and high pH values affected the average diameter of the emulsions prepared by MC. When Tw20 was used as an emulsifier, the emulsion was stable under neutral and weak alkaline conditions. The size and CV of the droplets did not change at different pH values and the emulsion was stable, indicating that pH had little effect on the emulsions prepared with Tw20.

However, the WPI- and SC-stabilized emulsions were both unstable at pH 3.0–9.0, especially near the isoelectric point (pH 4.0–6.0) of the two types of proteins. Therefore, they were only stable under neutral conditions. However, the mixed protein-stabilized emulsion was more stable at low and high pH levels, except at pH 4.0–5.0, which is close to the isoelectric points of the two types of proteins. This was because the stability of the natural proteins was greatly affected by pH; therefore, the WPI- and SC-stabilized emulsions tended to aggregate at lower or higher pH levels, resulting in instability or even the destruction of the emulsion droplets. However, the mixed protein emulsifier may overcome the defect of the isoelectric point, reduce the aggregation of the emulsion droplets, and further improve the stability of MC emulsions at lower or higher pH levels (33, 34).

#### Influence of Ionic Strength on Emulsion Stability

**Figure 9:**
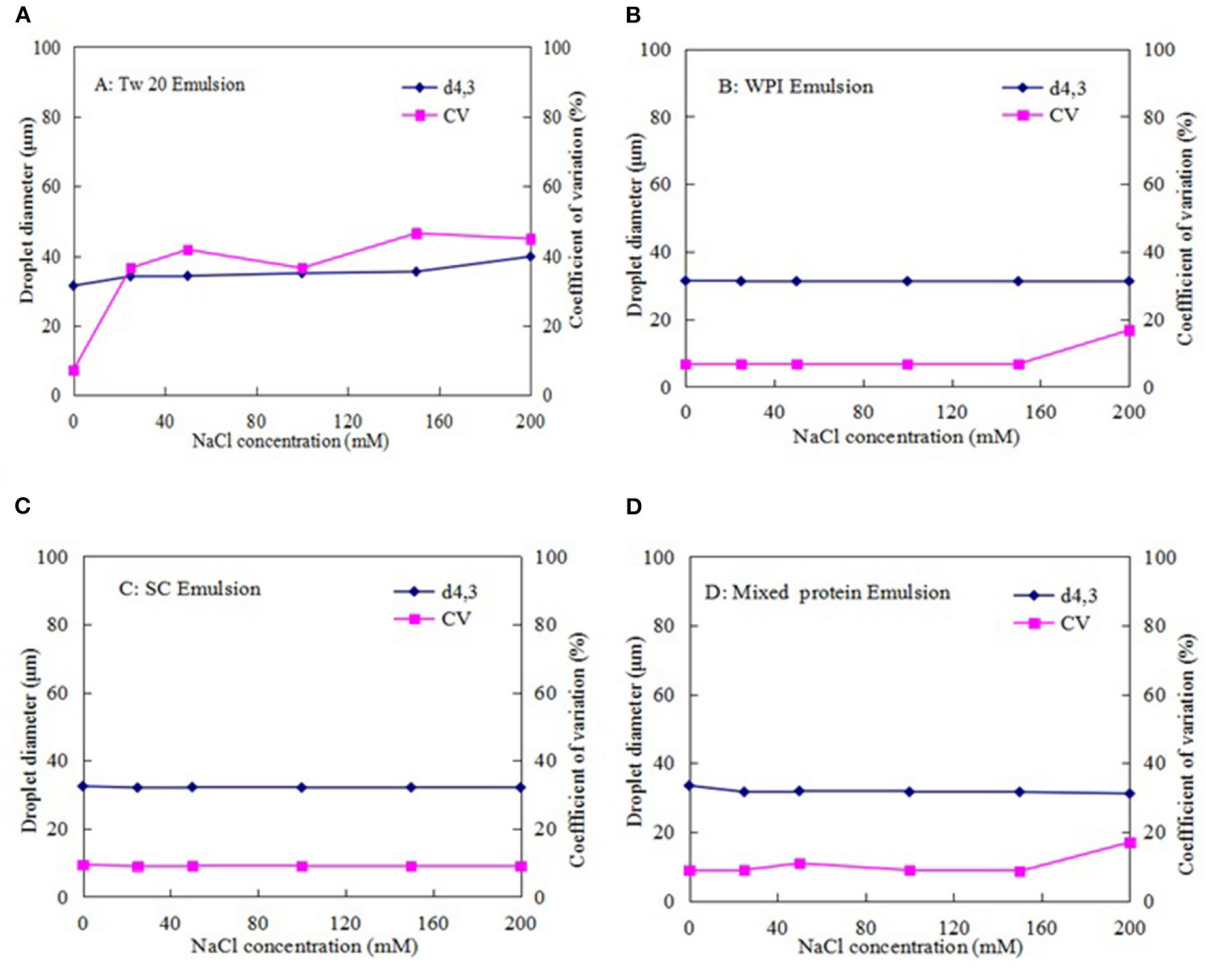
Effect of ionic strength on the stability of the MC emulsions stabilized by: **(A)** (Tw20), **(B)** WPI, **(C)** SC, and **(D)** mixed proteins.

Figures 9A–D shows that NaCl affected the average diameter of the emulsion droplets stabilized with Tw20. As the salt concentration was increased, the droplet size and CV were gradually increased, indicating that Tw20 and its emulsion are greatly affected by ionic strength.

In contrast, the SC-, WPI-, and mixed protein-stabilized emulsions were less affected by low salt concentrations (0–150 mM). When the NaCl concentration was increased to 200 mM, the average diameter and CV values of the emulsions stabilized by WPI and SC were increased. This could be attributed to aggregation and denaturation at high salt concentrations and resulted in an increase in the CV values of their MC emulsions (35). Nevertheless, the mixed protein emulsions were less affected by the high salt concentration, which was more stable than the single protein (SC or WPI)-stabilized emulsions.

#### Storage Stability of Emulsions

**Figure 10:**
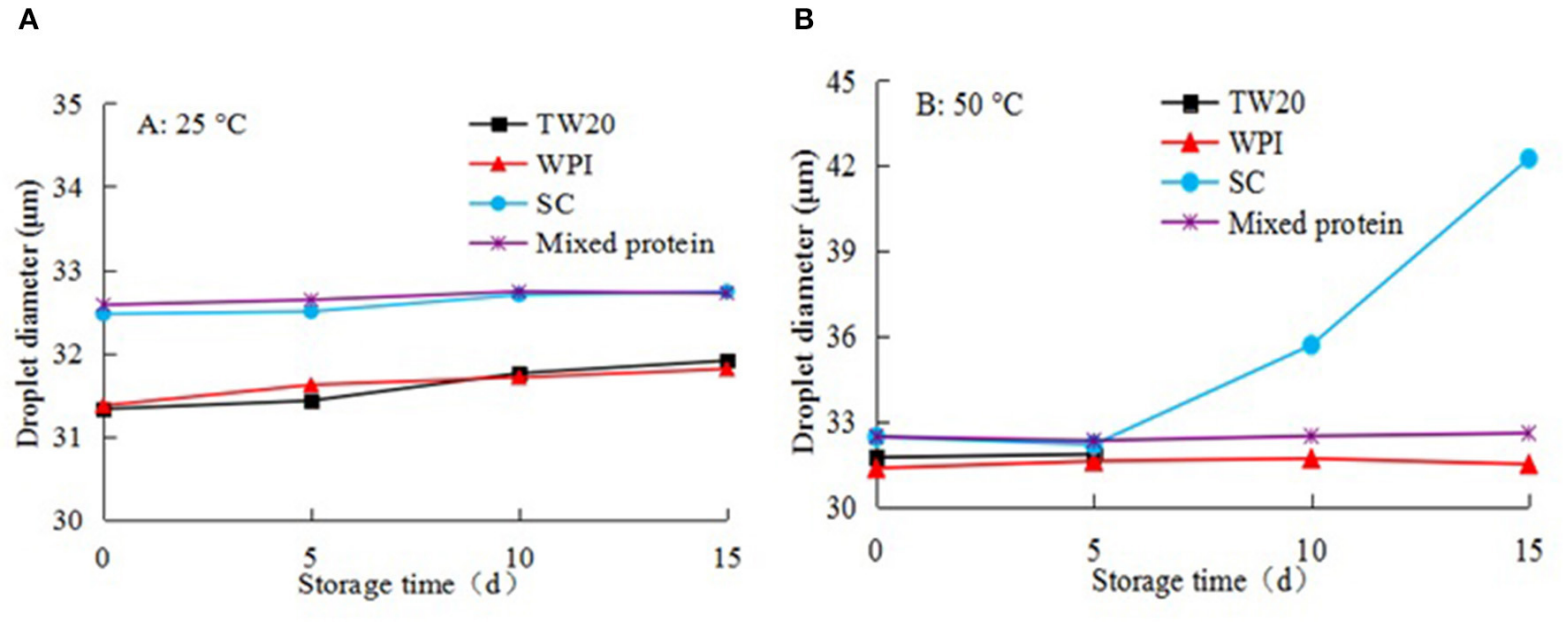
Storage stability of different microchannel (MC) emulsions at different temperatures: **(A)** 25°C and **(B)** 50°C during 15 days storage.

Figures 10A,B shows the physical stability of emulsions stabilized by the five types of emulsifiers over 15 d of storage at 25 and 50°C. During storage at 25°C, the droplets remained almost constant in the emulsions for 15 d, their size did not change significantly, and the emulsions remained stable. However, when the emulsions were stored at 50°C, the emulsions stabilized by Tw20 coalesced after 5 d, and the emulsion droplets stabilized by SC were also increased significantly.

In contrast, the emulsions stabilized by WPI and the mixed proteins were physically stable against droplet growth and were more stable than the emulsions stabilized by SC alone and Tw20. Therefore, the mixed SC and WPI emulsifier had better stability during long-term storage at 25 and 50°C, the MC emulsion can remain stable for a long storage life at room and medium temperatures.

This may be because the structures of WPI or SC were easily destroyed under high-temperature storage conditions; in particular, the spherical SC-stabilized emulsion was not stable. When SC was mixed with WPI to emulsify soybean oil, the denaturation temperature of the mixed emulsifier was increased, and the combination of the compound emulsifier and oil was enhanced at high temperatures, thereby, protecting the MC emulsion against heat-induced flocculation under high temperatures. Further evidence also supports the positive effect of SC and WPI on the thermal stability of the emulsions.

## Conclusions

Microchannel emulsions stabilized by two natural protein emulsifiers were successfully prepared. The results showed that relatively low levels of WPI (0.25–0.75%) in combination with low levels of SC (0.75–0.25%) can be used to form stable monodisperse O/W emulsions with a narrower droplet size distribution, larger droplets, and higher ζ-potential due of the improved emulsifying properties of the mixed proteins.

The MC emulsion also had better thermal stability and pH stability. The instability of a single protein under temperature and pH changes may be resolved by combining two proteins to confer the MC emulsions with better stability against environmental stresses than emulsions stabilized by a single protein emulsifier. The mixed protein emulsifier was more uniform and stable and could more effectively adsorb to the surface of the oil-water interface, reduce the aggregation of the emulsion at high temperatures and lower pH levels, and further improve the stability of the MC emulsion. At low salt concentrations, the mixed protein emulsions had better stability than the Tw20 emulsion.

All of the MC emulsions were stable during 15 d of storage at room temperature (25°C). The MC emulsions stabilized by Tw20 and SC underwent detectable coalescence after 5 d at 50°C; however, the MC emulsions prepared by the mixed WPI/SC protein emulsions were more stable at 50°C for 15 d than those emulsified by Tw20 and the single protein emulsifier. The mixed WPI/SC protein exhibited excellent ability for stabilizing the MC emulsion at medium temperatures, which was attributed to the thicker interfacial layer.

In summary, this study demonstrated that the MC emulsion stabilized with the mixed protein emulsifier had better properties and stability than the emulsions stabilized by the single protein and Tw20 emulsifiers. Next, we will further study the relationship between the binding mechanism and emulsifying properties of the mixed protein emulsifier. This study provides a novel research direction for developing natural emulsifiers for MC emulsions and increasing the utilization of MC emulsions in the food and pharmaceutical industries.

## Data Availability Statement

The raw data supporting the conclusions of this article will be made available by the authors, without undue reservation.

## Author Contributions

YJ: investigation, data curation, and writing the original draft. YZ: investigation. YC: writing review. ZM: data curation and writing review. MN: conceptualization, supervision, writing review, and editing. IK: validation and data curation. MAN: conceptualization and project administration. All authors contributed to the article and approved the submitted version.

## Funding

This research was partially funded by the program for promoting Grant-in-Aid for Scientific Research Applications FY 2021 from the Faculty of Life and Environmental Sciences, University of Tsukuba (Recipient: MAN). The authors IK and MAN are equally thankful to the Japan Society for the Promotion of Science (JSPS) for the financial support through Grants-in-Aid for Scientific Research KAKENHI (B) (Grant No. 21H008138).

## Conflict of Interest

The authors declare that the research was conducted in the absence of any commercial or financial relationships that could be construed as a potential conflict of interest.

## Publisher's Note

All claims expressed in this article are solely those of the authors and do not necessarily represent those of their affiliated organizations, or those of the publisher, the editors and the reviewers. Any product that may be evaluated in this article, or claim that may be made by its manufacturer, is not guaranteed or endorsed by the publisher.
